# Pilot Site Deployment of an IoT Solution for Older Adults’ Early Behavior Change Detection †

**DOI:** 10.3390/s20071888

**Published:** 2020-03-29

**Authors:** Hamdi Aloulou, Mounir Mokhtari, Bessam Abdulrazak

**Affiliations:** 1 ReDCAD, Centre de Recherche en Numérique de Sfax, Sfax 3021, Tunisia; 2 Direction de la Recherche, Institut Mines Télécom, 91120 Paris, France; 3 AMI-Lab, Université de Sherbrooke, Sherbrooke, QC J1K 2R1, Canada

**Keywords:** internet of things, pilot site, behavior change, frailty

## Abstract

The world demography is continuously changing. During the last decade, we noticed a regular variation in the world demography leading to a nearly balanced society share between the young and aging population. This increasing older adult population is facing many problems. In fact, the transition to the aging period is associated with physical, psychological, cognitive, and societal changes. Negative behavior changes are considered as indicators of older adults’ frailty. This is why it is important to detect such behavior changes early in order to prevent isolation, sedentary lifestyle, and even diseases, and therefore delay the frailty period. This paper exhibits a proof-of-concept pilot site deployment of an Internet of Thing (IoT) solution for the continuous monitoring and detection of older adults’ behavior changes. The objective is to help geriatricians detect sedentary lifestyle and health-related problems at an early stage.

## 1. Introduction

Aging is often related to significant changes in physical activities, mobility, nutrition, social life, and cognitive status. These changes considerably affect older adults’ quality of life. According to the World Health Organization (WHO) [1], the biggest health risk for seniors is the adoption of a sedentary lifestyle that causes isolation, depression and many other diseases such as cardiovascular disease, obesity, high blood pressure, etc.

In this paper, we present our pilot site deployment of an Internet of Things (IoT) solution for the continuous monitoring and detection of older adults’ behavior changes. The objective is to help geriatricians detect sedentary lifestyle and health related problems at early stage, without the need to perform classical psycho-geriatric tests that have many limitations like assessment inaccuracies and the difficulty for older adults to recall past events. The work was performed as part of the European project City4Age based on six pilot sites: Athens, Birmingham, Lecce, Madrid, Montpellier, and Singapore—each of them focusing on a specific topic: Athens (Social interactions through community centers), Birmingham (Public E-Services and digital technologies), Lecce (Daily activities in public social areas), Madrid (Mobility in the city and public transport), Montpellier (Indoor/outdoor assistive services for aging well), and Singapore (social activity and engaged community within the HDB). In this paper, we will focus on and detail the deployment performed in the pilot site of Montpellier.

## 2. Literature Review and Related Work

Early detection of ageing people behavior change can improve medical assessments and enable proactive intervention [2]. In fact, aging-related health problems generate long-term behavior changes, such as possible instabilities, variations, impairments, declines, increases, or improvements [3]. Nowadays, geriatricians use psycho-geriatric scales and questionnaires to analyze behavior and investigate possible changes. These questionnaires include Short Emergency Geriatric Assessment (SEGA) [4], Mini Mental State Examination (MMSE) [5], Geriatric Depression Scale (GDS) [6], Instrumental Activities of Daily Living (IADL) [7], Autonomie Gerontologique et Groupes Iso-Ressources (AGGIR) [8], and many others. These psycho-geriatric approaches are insufficient to monitor patients on a daily basis [9]. Thus, geriatricians need technological services to acquire new objective observations that complete their medical observations [10].

Monitoring technologies can help follow-up older adults at home and in the city, in order to early detect possible health changes [11]. During the last few years, ICT and IoT technologies have been largely used to monitor and follow older adults in their daily routines. For example, in [12], authors proposed a solution for continuous vital signs’ monitoring using different kinds of biomedical sensors. In another work [13], these technologies have been used for activity recognition in multiple residents’ homes. Similarly, the work in [14] proposed a technique for activity recognition based on acoustic events happening in daily life environments. However, systems proposed in these works are offering solutions for providing real-time and punctual measures and inferred activities without special interest on the evolution overtime and end-users’ behavior change. Some other works have investigated changes and anomalies for specific parameters and activities: The work in [15] was interested in studying and investigating the concern about falling for people with peripheral neuropathy (PN). Another work [16] developed a wearable device for monitoring people suffering from muscle disorder. Paragliola and al. [17] proposed a solution for gait anomaly detection of Parkinson users based on machine learning techniques. In [18], authors propose a method for motion disorders detection of patients with autism spectrum disorders using a sensor worn on the wrist. The systems proposed in these cases are dedicated to some specific scenarios and use-cases and are not for generic use by elderly people. There exist some works that have investigated behavior change and anomaly detection over the time [19,20,21]; however, these works have not carried health and medical correlation with the detected changes and anomalies in order to take appropriate interventions.

Regarding research works that have investigated medical and health related behavior change, we can distinguish four main categories: The first category targets “Short-term Health Change Detection” occurring in specific time periods, such as falls and wandering during sleep [22,23]. The second category retrospectively investigates possible changes after change occurrence [24,25]. The third category compares older adults’ populations with or without a target disease, in order to investigate disease-related indicators at population scale. Existing studies investigate indicators of mild cognitive impairments (MCI) [26,27]. The last category uses intrusive technologies to capture video sequences, collect daily questionnaire-based information and record physical data using body sensors [28,29,30,31,32].

Contrary to these approaches, our proposed approach analyzes overall behavior over long periods, in order to detect long-term changes in health status. These long-term changes require weeks and months to emerge, and are difficult to detect due to normal continuous variation in human behavior [33]. In addition, it continuously analyzes monitoring data on a daily basis, in order to early detect possible changes. This proactive change detection provides opportunity for daily assessment and subsequent intervention. Moreover, it analyzes older adults’ behavior at temporal scale, in order to detect person-centered changes compared to past habits. This allows for providing individual assessment and personalized intervention after detecting possible changes. Finally, it uses unobtrusive monitoring technologies that are embedded in our real environment or in objects of daily living, do not affect privacy, do not interfere with natural behavior of older adults, and do not change their daily habits.

## 3. Montpellier Pilot Setup

Montpellier pilot site goal is to quickly and unobtrusively detect possible aging people’s behavior changes. Detected behavior changes are analyzed afterwards and confirmed by collaborating geriatricians to provide adequate intervention [34].

The pilot site is coordinated by the French National Center for Scientific Research (CRNS) and is collaborating with local authorities such as Montpellier Metropolis, the ETAPE association (Health Autonomy Pole), and healthcare professionals from Beausoleil Clinic and Saint Vincent De Paul nursing home.

**Figure 1:**
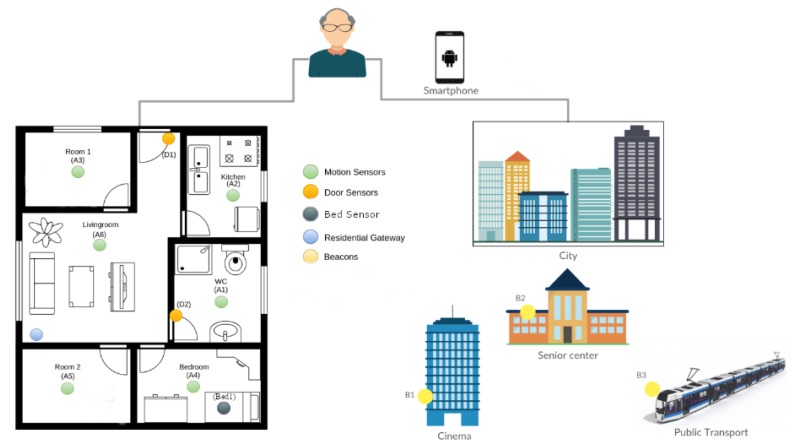
Montpelier pilot site global setup.

The proposed solution deployed in the pilot site of Montpellier consists of a set of sensors deployed in the participants’ homes and in the city as shown in Figure 1. For indoor monitoring, the pilot uses motion sensors, contact sensors, and bed sensors. Outdoor monitoring is based on Smartphones carried by participants and beacons deployed in places of interest of participants in the city. These sensors allow for collect raw data and objective information in real time, which are processed to detect behavior changes.

## 4. Recruitment and Engagement

Each of the six pilot sites followed a specific ethical approval process, related to specific countries’ regulation, in order to recruit participants. For the Montpellier pilot site, the ethical process consists of submitting an application to the Institutional Review Board (Comités de Protection des Personnes: CPP).

**Figure 2:**
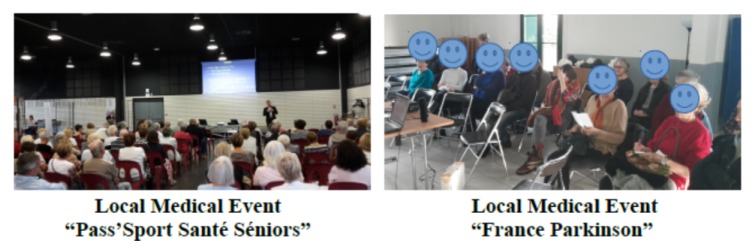
Briefing and involvement sessions.

After obtaining the ethical approval, we started the recruitment process. We have approached around 40 potential participants with the help of ETAPE association and Saint Vincent De Paul nursing home. In fact, we have presented the solution in several local events to promote the project and identify interested people to be included in the study. As shown in Figure 2, briefing and presentation sessions were organized in the Montpellier pilot site to better promote experimentation objectives, quickly launch the recruitment process, and keep recruited participants involved in the experimentation. These events allowed for having in-depth discussions with interested aging people who accepted to visit a demonstration house, see a live demonstration of the system, and get their feedback. The demonstration house highlights the unobtrusiveness of the technological solution proposed for the potential participants. Interested people also observe sensor events in real-time and examples of real data over weeks and months indicating significant changes in health status. Potential participants asked for information about employed technological solutions, real benefits of adopting them at home, and possible risks.

Nineteen participants accepted and have been equipped with the City4Age solution. An initial interview with included participants allowed for collecting some indications on their social and health profiles. Participants have diverse medical and social profiles such as educational level, dependence level, habits, and health status. Table 1 summarizes the social profiles of the different participants. All recruited participants were over 60 years old and living alone (11% Divorced, 5.5% Single, and 83.5% widow). In addition, 72% of them were Female (28% were Male) and 89% receive medical or social care at home. Table 2 presents regular habits and health info of some participants. Recruited participants have diverse daily and weekly habits, and medical profiles. We were interested in some specific habits such as wake-up time in the morning, toilet entries, eating time, watching television time (preferred activity for many elderly people), family and caregivers visits, etc. First, interviews with elderly people, their family members and formal caregivers investigate health status at the starting time of the monitoring period. Initial medical profiles and health status diagnosis validate participant recruitment, notify about diverse physical and cognitive problems, and provide primary explications for future detected health changes.

## 5. Technologies, Data Collection, and Data Analysis

The system proposed in the Montpellier pilot site uses indoor and outdoor technologies to monitor daily living activities of participants. For indoor monitoring, the pilot is proposing a set of sensors (motion sensors, contact sensors, and bed sensors). Outdoor monitoring is based on beacons deployed and tagging specific places in the cities considered as participants’ places of interest (e.g., bus and metro stations, cinemas, restaurants, etc.). The communication between beacons and the pilot site local server is guaranteed through a dedicated mobile application installed on the the participants’ smartphones. The characteristics and usage of the sensors deployed in the pilot site is detailed in Table 3.

**Figure 3:**
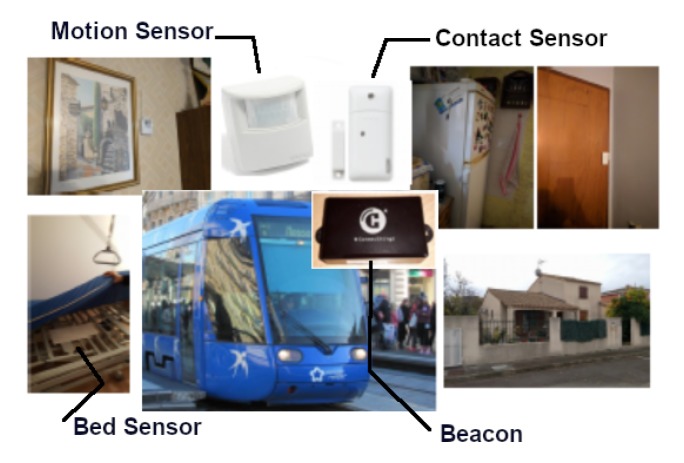
Indoor and outdoor sensors’ deployment.

These sensors operate discreetly and allow for collect raw data and objective information in real time indoors and outdoors in the city. The objective is to be able to accurately determine the “habitual behaviors” of people by collecting data over time. Figure 3 showcases some deployed sensors in the Montpellier pilot site. For ethical reasons, the collected data will be available on request.

**Figure 4:**
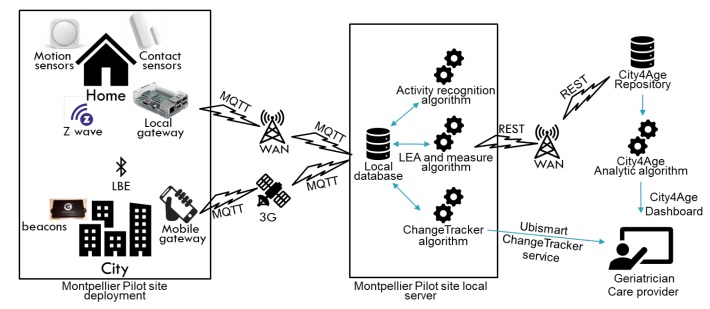
Complete architecture of Montpellier pilot site’s deployment.

All indoor and outdoor data are collected in the Montpellier pilot site local server. Data are completely anonymous and are referred uniquely by a unique participant identifier. The correspondence between the participant identifier and his identity is only accessible to the pilot site coordinator when an investigation or an intervention is needed. On the pilot site local server, collected data are used for the identification of Low Elementary Actions (LEAs) and measures. Low Elementary Actions are basic participants’ actions which are inferred from received sensors’ events (e.g., Start Moving, Stop Moving, Change Room, Visit Restaurant, etc.). Measures are quantified data extracted from LEAs (e.g., time in the bedroom/day, number of toilet visits/day, number of shops visits/week, etc.). Later, this information is transferred to the City4Age repository and analytic algorithm where further treatments are performed in order to produce visualizations for the geriatrician. The complete architecture of the City4Age solution and the performed deployment and data analysis in the Montpellier pilot site is presented in Figure 4.

We have identified relevant LEAs Based on internationally-validated geriatric references (e.g., SEGA [4], MMSE [5], GDS [6], IADL [7], AGGIR [8], and others) and distinguished four main categories that can be monitored by our system: activities of daily living, mobility, social life, and nutrition as detailed in Table 4. LEAs were recognized using the semantic modeling and reasoning system we have proposed in [35].

LEAs were quantified based on time, place, number, and duration metrics in order to produce measures. These measures were analyzed by our change detection algorithms to detect possible behavior changes (e.g., sleep interruption number increases during anxiety periods, go shopping frequency decreases because of mobility impairments, toilet entries increase due to urinary infection, and preparing meal duration increases because of cognitive problems). Table 5 introduces the used metrics with some examples while the list of measures collected in Montpellier pilot site is presented in Table 6.

The deployment of our proposed solution in France allowed for collecting around two years of real data. We have collected 310,590 of Low Elementary Actions (LEA) and 49,659 of Measures for 19 participants.

## 6. Behavior Change Detection

**Figure 5:**
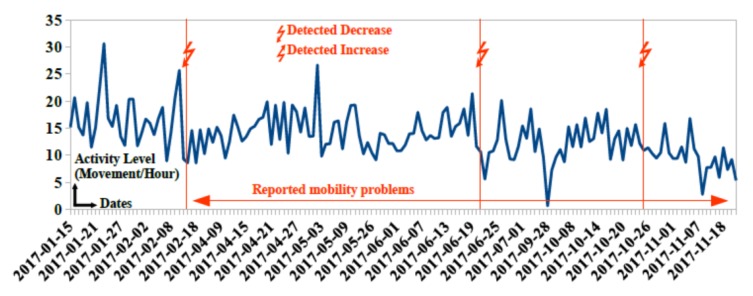
Detected changes in activity level of participant 91 due to mobility impairments.

**Figure 6:**
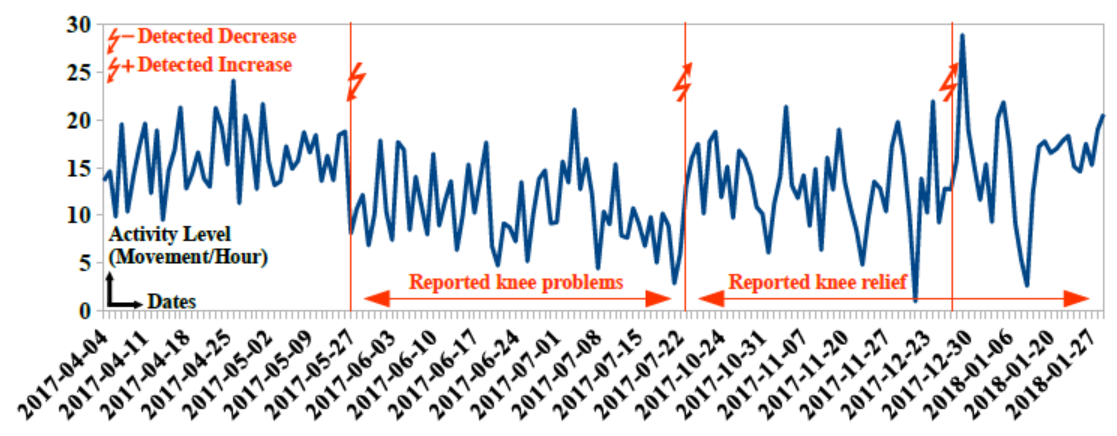
Detected changes in activity level of participant 99 due to knee problems.

The goal of the system proposed in Montpellier pilot site is to detect possible behavior changes that will be analyzed and confirmed by collaborating geriatricians to provide adequate intervention. A behavior change tracker service “ChangeTracker” [36] was developed allowing for detecting changes in participants’ behavior using statistical algorithms. Collected data are analyzed by our “ChangeTracker” service and presented to collaborating geriatricians from Beausoleil clinic. Possible behavior changes are detected 2 to 10 days after change occurrence. Figure 5 and Figure 6 showcase some behavior changes detected by the “ChangeTracker” service for some participants in the pilot site.

In Figure 5, three consecutive decreases on 2017-02-15, 2017-06-20 and 2017-10-25 are detected for participant 91. Participant and family doctor confirm mobility impairments and increased risk of dependence in managing activities of daily living. Professional caregiver helps with medication taking and household from 2017-05-04.

In Figure 6, a first detected decrease occurs on 2017-05-27. Nurses report knee problems in 2017-06. Observed mobility impairments reduce activity level of participant 101. After treating knee problems, the “ChangeTracker” service detects two consecutive positive increase in activity level on 2017-07-23 and 2017-12-24. Nurses observe considerable improvement in physical health status in 2017-09.

## 7. Intervention Process

To perform intervention, the pilot site is providing a framework with visualizations about participants activities and statistics about their daily routines and habits. The “ChangeTracker” service is integrated in this framework allowing to automatically detect possible changes that can be confirmed by the caregivers and the geriatrician. The caregivers and geriatricians can navigate the data provided by these visualizations and decide on the type and form of intervention when needed. Below are some examples of interventions decided by the medical and caregiver staff:

- After detecting a decrease in outdoor and indoor activities for participant 96, nursing home stuff decided to initiate home assistance
- Detecting a decrease in outdoor activities for participant 92 allowed the geriatrician to decide on the hospitalization of this participant.
- Detecting decrease in toilet visits for participant 94 and an increase in activity level for participant 95 allowed the geriatrician to change the medical treatment for these two participants

## 8. Validation

In this paper, we will focus on the technical validation of the pilot site setup and the performance evaluation of the developed “ChangeTracker” service based on medical observations and collected health records.

### 8.1. Technology Validation

**Figure 7:**
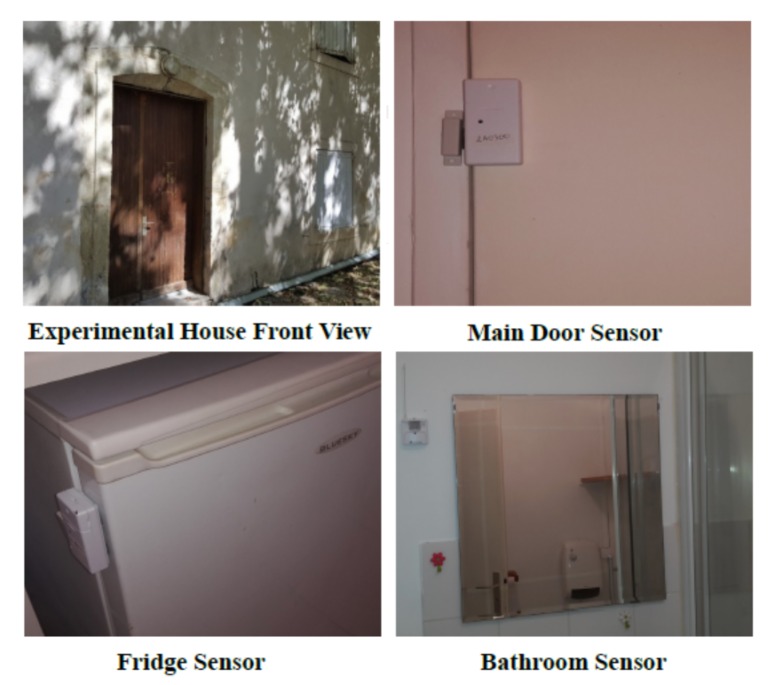
Montpellier pilot site demonstration house.

We have firstly validated the selected sensors and devices that have been used in the deployment. In the Montpellier pilot site, we have deployed a demonstration house as shown in Figure 7 and deployed outdoor sensors in the metropolis of Montpellier. All employed technologies, such as movement sensors, contact sensors, gateways, receivers, beacons, and Internet access points have been tested in this experimental house before being deployed. These experimentation allowed for identifying and fixing some technical problems in the technologies used.

The validation of collected data in Montpellier pilot site started with the data collected from the demonstration house. This phase allowed for validating the daily activity recognition process based on collected row data and real observations.

**Figure 8:**
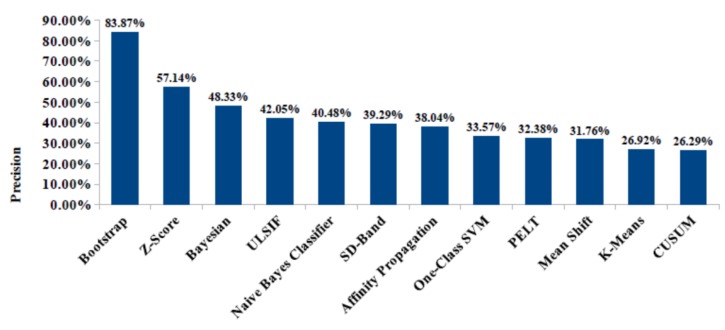
Precision of behavior change techniques evaluated by the “ChangeTracker”.

Later, we have integrated the “ChangeTracker” service to identify possible participants’ behavior changes. Different statistical, probabilistic, and machine-learning algorithms have been tested [37]. These change detection techniques do not provide the same performance in terms of change detection precision. In fact, change detection techniques do not detect the same change points for tested inferred data. Therefore, applying these techniques on real monitoring data for the same monitored participants enables effective performance evaluation. Correlating change points detected by our “ChangerTracker” service with medical observations and health records is essential to evaluate the used change detection techniques’ medical relevance. For this reason, we have calculated the change detection precision of the used change detection techniques. Change detection precision refers to the percentage of detected changes with retrieved medical explication (i.e., changes related to health status based on medical correlations) from the global detected changes. Unexplained changes could be related to different reasons such as false alarms or undiagnosed health problems (e.g., participant wakes-up earlier, but does not feel this change and does not find explication). Figure 8 shows measured precision for investigated change detection techniques. Bootstrap technique provides best precision of 83.87%; i.e., bootstrap technique detects significant changes that are highly related to physical and health problems.

### 8.2. Detection Process Validation

**Figure 9:**
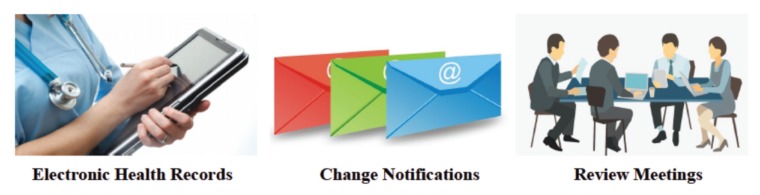
Correlation of detected changes with medical observations.

The “ChangeTracker“ service has been validated with the help of our collaborating geriatrician. Detected changes have been correlated with medical observation and health records to validate the used algorithms and their performances as showcased in Figure 9.

The “ChangeTracker” service sends change notifications from 2 to 10 days after change occurrence. These notifications inform about change date, monitored participant ID, and means before and after detected changes.

**Figure 10:**
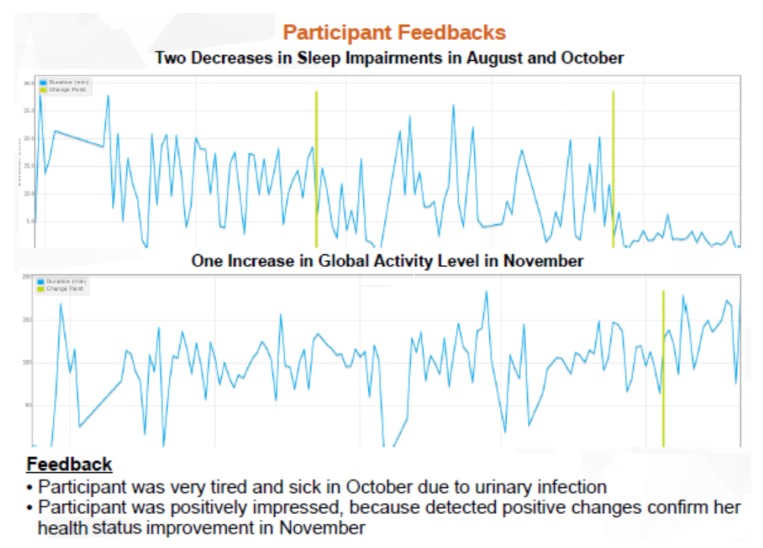
Detected behavior changes by “ChangeTracker” and corresponding participant feedback.

Change notifications provide an opportunity to confirm that detected changes are really permanent and investigate possible correlations with geriatric observations. Regular review meetings with older adults, family members, and family doctors allow for accurately investigating possible causes of detected changes. The pilot site local geriatrician evaluates the medical relevance of investigated change explications, by reviewing all past detected changes and correlating them with medical records (e.g., geriatric scales, cognitive diagnosis, and prescribed treatments). Review meetings investigate multi-dimensional correlations of detected changes, such as identifying parallel decreases in activity level and time out home related to mobility impairments, and consecutive increases in sleep interruptions and toilet entries after treatment change. Figure 10 shows some results of changes detected by “ChangeTracker” service and their correlation with medical observations and participant feedback.

Evaluating medical relevance of detected changes requires continuous collection of personal and medical information. Personal interviews with monitored older adults enable investigation of possible causes explaining observed changes; e.g., detected changes in activity patterns correlate with personal feelings of slowness in physical movements and trembling in fingers related to Parkinson’s, and detected changes in preparing meal duration correlate with forgetting recent events related to cognitive impairments. Regular review meetings allow for accurately investigating possible causes and investigating multi-dimensional correlations. Validating data collection with older adults, family members, and family doctors provides better correlation of detected changes in monitoring data with real medical health status. Professional caregivers collect electronic health records while visiting monitored participants a couple of times per day for medicine taking, toilet entry assistance, room cleaning, and nutritional services. They report all interventions, formal and informal observations, special health events, and social habits.

### 8.3. Results and Performance

**Figure 11:**
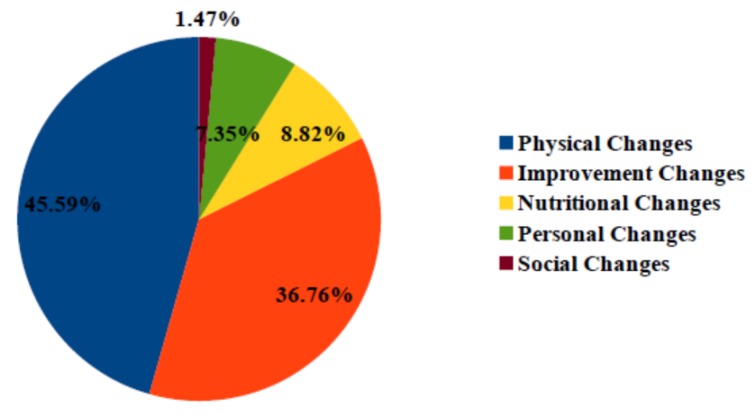
Possible cause rates of detected changes in individual houses.

In total, our “ChangeTracker” service detected 340 changes for all participants with an average of 0.97 change per month. Participants show diverse changes in monitoring periods that have been correlated with diverse reasons, such as physical problems, health improvements, nutritional problems, personal changes, and social problems as detailed in Figure 11.

Health improvements justify 36.76% of detected changes; e.g., participant 171 has fewer sleep interruptions correlated with improvement in sleep habits and toilet entries. Physical health problems present main detected change cause for individual house participants with a percentage of 45.59%; e.g., participant 95 sleeps longer with fewer interruptions correlated with Parkinson freezing periods. Nutritional, personal, and social changes influence monitored health status with respective rates of 8.82%, 7.35%, and 1.47%; e.g., participant 91 receives less visits at home due to social isolation.

### 8.4. Health Change Detection Ontology

**Figure 12:**
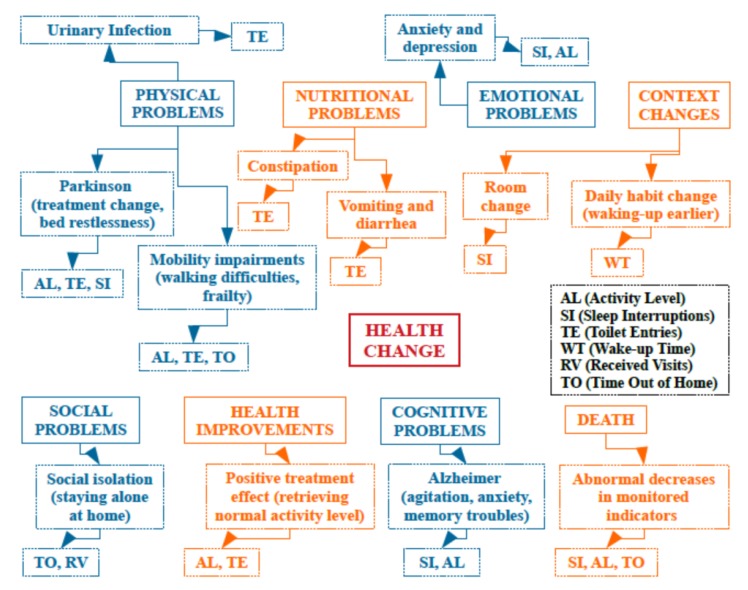
Health change detection ontology.

Based on the realized work, and the collected medical interpretations of detected changes in all monitored health change indicators (e.g., activity level, sleep interruptions, toilet entries, received visits and time out of home), we have defined a global health change detection ontology, presented in Figure 12, that can be used to identify possible cause categories and sub-categories for detected health changes. Medical interpretations have been performed using records, personal interviews, and regular review meetings with elderly people and family members. The main health change causes that have been identified are physical problems, nutritional problems, emotional problems, context changes, social problems, health improvements, cognitive problems, and death. This ontology can be later extended and populated with more observations and interpretations.

### 8.5. Stakeholders’ Feedback

Participants show considerable interest in early health change detection and more acceptance for unobtrusive monitoring. A positive feedback was collected from the participants and the different stakeholders. Below is some feedback collected from participants, geriatricians, and caregivers:

- **Participant:** I’m happy to participate in this research. Sensors do not bother me at all. They are now part of my house. I do not think about them. The results with the way we quantify my indoor movements and my activities are interesting.
- **Geriatrician:** We are working with patients with Parkinson’s disease. In this special disease, there are many problems concerning sleep and voiding function, and we have a solution to propose to them. However, in short consultations, we don’t have time to speak about all things and we know very few things about patients’ activities of daily living. We think that an unobtrusive technological solution will be interesting to help us to improve our assessment.
- **Caregiver:** My mother is participating in the project. The system doesn’t affect privacy. This is very important, and our feedback is positive. We could detect changes that correlate with my mother’s health status. This was beneficial for our discussions.

## 9. Conclusions

The Montpellier pilot site was a proof-of-concept of the solution proposed by the City4Age project. The pilot site realized the necessary tasks from ethical approval, building local partnerships, and deploying the proposed solution. It allowed to put in place a mechanism for the early identification of behavior changes and the provision of adequate intervention. In total, 340 changes have been detected for all participants. These changes have been validated and classified with the help of a local geriatrician and by correlation with Medical Observations. In the Montpellier pilot site, the economic value of the solution proposed by City4Age has been identified on different aspects. First, this solution could be of great value for the geriatricians, and this was confirmed by the testimony from our local geriatrician. In fact, the technological observations provided by City4Age solution enrich their medical observation for better assessments of frailty and MCI. The solution is also valuable for nursing homes. In fact, nursing homes need to remotely receive reliable information on health status, in order to offer personalized healthcare services for older adults. It requires maintaining independent living at home and identifying older adults at risk who really require entry to nursing homes. Finally, the solution can be proposed to interested aging people through public non-profit organizations like ETAPE in Montpellier. This organization provides free services for older adults to help them adapt their living environment to their needs.

## Figures and Tables

**Table 1:** Montpellier pilot site participants’ social profile.

Id	Birth Date	S	Marital Status	Education	Care
91	1934	F	W	primary	Yes
92	1949	M	D	secondary	Yes
93	1939	F	W	tertiary	No
94	1956	F	D	tertiary	Yes
95	1959	M	S	tertiary	Yes
96	1923	M	W	secondary	Yes
97	1923	F	W	primary	Yes
98	1925	F	W	none	Yes
99	1926	F	W	none	No
100	1928	M	W	secondary	Yes
101	1928	F	W	none	Yes
102	1932	F	W	secondary	Yes
103	1929	F	W	secondary	Yes
170	1928	F	W	secondary	Yes
171	1927	F	W	secondary	Yes
172	1922	F	W	secondary	Yes
173	1919	F	W	secondary	Yes
174	1933	M	W	secondary	Yes

**Table 2:** Montpelier pilot site participants habits and health status.

Patient	Regular Habits	Health Info
91	Wakes up at 7 h. Goes to toilet. Takes breakfast. Goes out for 1 hour to take care of animals. Goes out between 12 h 30 and 14 h for lunch with his daughter. Reads newspapers. Frequently goes out during the day. Friend visits on Sundays midday. Goes out shopping Wednesdays.	Very active person. No special diseases. Recent mobility impairments. Recent social isolation. Recent nutritional problems.
98	Wakes up at 8 h. Home aid 4 times per day. Stays most often at home. Sometimes goes out with daughter or caregiver.	Alzheimer. Diabetes. Vision and audition problems.
101	Wakes up at 7 h 30–8 h. Home aid visits 3 times per day (morning, midday and evening). Niece and neighbor visits during the day. Sleeps earlier than before (at 20 h, and before at 22 h).	Alzheimer. Some falls and hospitalizations.
102	Wakes up at 6 h–7 h. Home aid visits each day in the morning. Lives alone. Daughter house is nearby. Monthly visits to and from daughter.	Heart problems. Urinary infection.

**Table 3:** Deployed sensors’ characteristics and usage.

Technology	Model	Raw Data	Inferred Data	Number	Location
Movement sensor	Z-wave MultiSensor	Presence/absence of movements	Walking patterns, received visits, sleep interruptions, toilet entries	4–5/part	One sensor/room
Contact sensor	Z-wave Door/Window Sensor	Openings/closings of objects	Come home, go out, prepare meal, take medicines, read books	3–4/part	On specific objects
Bed sensor	Fiber optic bedsensor	Bed movements, heart beats, respiration rate, rhythm, depth	Sleep time, wake-up time, sleep duration, bed restlessness, cardiac events, respiration	1/part	On the bed
Beacon sensor	BLE beacon Sensor	Unique identifier bound to specific locations in the city	Shops visits, restaurants visits, cinema visits, transport usage	4–5/part	Attached in specific locations in the city

**Table 4:** Low Elementary Actions’ categories.

Category	Sub-Category	Examples	Relevance	Technology
Activity of Daily Living	House activities	Clean, tidy-up rooms, reading, watching TV, put laundry, wash dishes	Physical, cognitive impairments, autonomy loss	Door, movement
	Upper hygiene	Shave, dress one’s hair		
	Inferior hygiene	Hygiene of intimate, inferior members, legs, feet, nails		
	Elimination	Urinary and fecal elimination		
Mobility	Moving	Between the rooms, to areas of interest in the city	physical problems	beacons, movement, door
	Position changes	Walk, get up, turn around, sit		
Social Life	Go out	Use means of transport, shopping, free time activities	Social isolation	beacons
Nutrition	Eat	Protein, fruit, vegetable	digestive problems, depression	movement, door

**Table 5:** Metrics for measures’ calculation.

Metric	Description	Examples
Time	Start and end times of executing monitored activities	eating time, sleep time, wake up time, watch TV time
Place	Where monitored activities are executed	shopping place, entertainment place, physical activities place cultural activities place
Number	quantity and amount of human activity execution	number of sleep interruptions, number of toilet entries, number of meals
Duration	length of executing monitored activities	sleep duration, watch TV duration, out of home duration

**Table 6:** Montpellier pilot site measures.

Category	Collected Measures	Periodicity
Indoor measures	NB_ROOM_CHANGES, NB_BEDROOM_VISITS, TIME_BEDROOM, NB_LIVINGROOM_VISITS, TIME_LIVINGROOM, NB_RESTROOM_VISITS, TIME_RESTROOM, NB_KITCHEN_VISITS, TIME_KITCHEN, NB_BATHROOMS_VISITS, TIME_BATHROOM, NB_MEALS, TIME_MEALS, TIME_HOME, TIME_OUTDOOR, NB_OUTDOOR, TIME_SLEEP	/day
Outdoor measures	NB_SHOPS_VISITS, TIME_SHOPS, NB_SUPERMARKET_VISITS, TIME_SUPERMARKET, NB_RESTAURANTS_VISITS, TIME_RESTAURANTS, NB_CINEMA_VISITS, TIME_CINEMA, NB_PHARMACY_VISITS	/week
